# Anti-inflammatory and antioxidant properties of betaine protect against sepsis-induced acute lung injury: CT and histological evidence

**DOI:** 10.1590/1414-431X2023e12906

**Published:** 2023-11-13

**Authors:** O. Sorgun, A. Çakır, E.S. Bora, M.A. Erdoğan, Y. Uyanıkgil, O. Erbaş

**Affiliations:** 1 Department of Emergency Medicine, Izmir Tepecik Training and Research Hospital, Izmir, Turkey; 2 Department of Emergency Medicine, Canakkale Mehmet Akif Ersoy State Hospital, Canakkale, Turkey; 3 Department of Emergency Medicine, Izmir Ataturk Training and Research Hospital, Izmir, Turkey; 4 Department of Physiology, Faculty of Medicine, Izmir Katip Celebi University, Izmir, Turkey; 5 Department of Histology and Embryology, Ege University, Izmir, Turkey; 6 Department of Physiology, Faculty of Medicine, Demiroğlu Bilim University, Istanbul, Turkey

**Keywords:** Acute lung injury, Betaine, Sepsis, Rat model

## Abstract

The aim of this research was to determine the anti-inflammatory effect of betaine on sepsis-induced acute respiratory distress syndrome (ARDS) in rats through histopathological examination, radiologic imaging, and biochemical analysis. Eight rats were included in the control group, and no procedure was performed. Feces intraperitoneal procedure (FIP) was performed on 24 rats to create a sepsis-induced ARDS model. These rats were separated into three groups as follows: FIP alone (sepsis group, n=8), FIP + saline (1 mL/kg, placebo group, n=8), and FIP + betaine (500 mg/kg, n=8). Computed tomography (CT) was performed after FIP, and the Hounsfield units (HU) value of the lungs was measured. The plasma levels of tumor necrosis factor (TNF)-α, interleukin-1β (IL-1β), IL-6, C-reactive protein, malondialdehyde (MDA), and lactic acid (LA) were determined, and arterial oxygen pressure (PaO_2_) and arterial CO_2_ pressure (PaCO_2_) were measured from an arterial blood sample. Histopathology was used to evaluate lung damage. This study completed all histopathological and biochemical evaluations in 3 months. All evaluated biomarkers were decreased in the FIP + betaine group compared to FIP + saline and FIP alone (all P<0.05). Also, the parenchymal density of the rat lung on CT and histopathological scores were increased in FIP + saline and FIP alone compared to control and these findings were reversed by betaine treatment (all P<0.05). Our study demonstrated that betaine suppressed the inflammation and ameliorated acute lung injury in a rat model of sepsis.

## Introduction

Sepsis is a pathological condition characterized by an aberrant immune response of the host to invading pathogens, resulting in hemodynamic alterations that can lead to severe multiple-organ dysfunction. Various etiological factors can cause this condition (1). The global prevalence of sepsis in hospitals and intensive care units is rising, and despite recent advancements in intensive care support technologies, a significant proportion of patients (30-45%) succumb to severe sepsis and septic shock following admission (2). The escalation in sepsis prevalence has resulted in significant loss of life and economic consequences, propelling research toward novel treatment modalities. The predominant therapeutic modalities presently employed include fluid resuscitation, administration of vasoactive agents, judicious use of antibiotics, immune modulation, and hemofiltration (3).

The lung is the organ most commonly and gravely impacted by the unregulated and disproportionate immune reaction of septicemia. Sepsis-induced acute lung injury (ALI) can advance to acute respiratory distress syndrome (ARDS), which is a crucial determinant of disease severity in septic patients (1). The clinical presentation of this condition includes hypoxemia, diffuse bilateral pulmonary infiltration, pulmonary edema, decreased pulmonary compliance, and reduced functional residual capacity. Due to alveolar-capillary membrane dysfunction, protein-rich fluid extravasation, alveolar hemorrhage, and fibrin deposition, there is an increase in the permeability of the blood vessels. These findings have been documented in the literature (2-4).

The activation of nuclear factor κB (NF-κB) induces the expression of diverse substances, including cytokines, chemokines, and nitric oxide (NO) (5). Prior research has demonstrated that sepsis can lead to the buildup of proinflammatory cytokines, such as tumor necrosis factor-alpha (TNF-α) and interleukin-1β (IL-1β), within the pulmonary system. These cytokines are known to have a significant influence on the pathogenesis of acute lung injury, as evidenced by previous research (6,7).

Due to inflammatory mechanisms, ARDS is characterized by the accumulation of water and cellular material in the alveoli, which are normally filled with air. However, it has been observed that fibrosis, which occurs during the fibro-proliferative phase of ARDS, results in a loss of aeration, leading to a higher radiological density of the lungs (8,9). The radiological visualization of interstitial edema, consolidation, and fibrosis in ARDS is most effectively achieved through computerized tomography (CT). Numerous studies have reported that the Hounsfield units (HU) scale of lung parenchyma density can serve as a predictive indicator of ARDS (10,11).

Betaine, a donor of methyl groups, holds significant physiological importance as an osmoprotectant (12). Betaine is a naturally occurring compound ubiquitous in animals, plants, and microorganisms. Additionally, betaine is endogenously synthesized from choline in the human body. The amino acid methionine has a crucial function in the antioxidant process. It is synthesized from homocysteine with the aid of betaine. Betaine functions as a methyl donor during the iterative transformation of homocysteine to methionine, when catalyzed by homocysteine methyltransferase in the hepatic tissue. Betaine has been found to have beneficial effects through its anti-inflammatory properties in various diseases, including obesity, alcoholic and non-alcoholic hepatosteatosis, diabetes, cancer, and Alzheimer's disease (13-17). In sepsis, free radical production increases in the lungs, and oxidative stress, responsible for organ dysfunction, occurs when the oxidant-antioxidant balance is disturbed.

ALI due to sepsis is common in emergency services, and effective and fast treatments are needed. While betaine's antiviral and anti-inflammatory properties are known, the underlying mechanism is unknown. The aim of this study was to determine the possible therapeutic effects of betaine in oxidative stress and inflammatory markers of ALI by evaluating lung parenchymal density changes on CT and verifying their correlation with histopathological findings.

## Material and Methods

### Animals

The study utilized 37 adult male albino rats (Wistar strain) weighing 200-250 g, obtained from the Experimental Animal Laboratory of Demiroğlu Bilim University (Turkey). All experiments complied with the regulations outlined in the Guide for the Care and Use of Laboratory Animals of the National Institutes of Health (USA). Before commencement, the Animal Ethics Committee of Demiroğlu Bilim University approved the study with reference number 27210519. The rats were allowed free access to food and water and were housed in standard cages maintained at 22.0±2.3°C. The rats were exposed to a daily cycle of light and darkness (12h/12h).

### Experimental procedures

**Figure 1:**
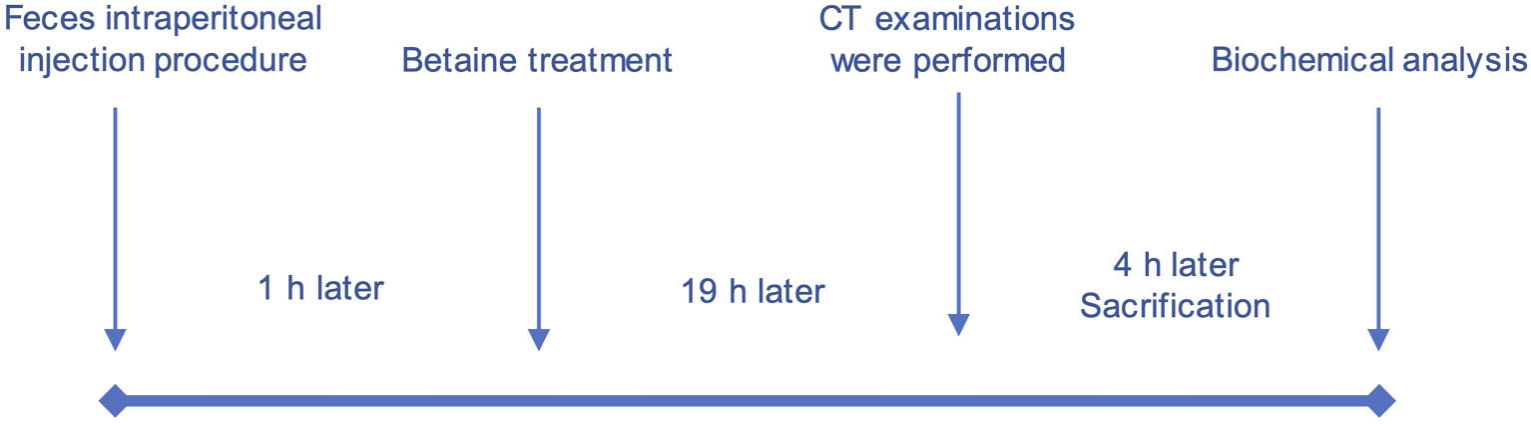
Timeline of the study.

The sepsis model was created by the feces intraperitoneal injection (FIP) method as described by Dibekoğlu et al. (18) and Canbolat et al. (19). For the FIP model, fresh feces were collected from rats (not included in this study) and suspended in saline at a concentration of 75 mg/mL. Then, the solution was injected with a 21-gauge needle intraperitoneally (*ip*) at 1 g/kg body weight only once. The research inquiry was completed within a 24-h duration. Rats were randomly assigned to four groups. Eight rats were included in the control group and received no FIP. FIP was performed to induce sepsis. In the first hour after FIP, animal groups were defined as follows: control group (no procedure, n=8); FIP group (sepsis, n=8); FIP + saline (0.9% NaCl) (n=8) at a dose of 1 mL/kg per day *ip*; FIP + betaine (at 500 mg/kg per day *ip*). Five rats died before the CT examination (within the first 20 h after FIP) and were excluded from the study (FIP group: 2 rats; FIP + saline group: 2 rats; FIP + betaine group: 1 rat). At the end of 20 h, thorax CT was performed under anesthesia. The study was completed 24 h after FIP. After all procedures, a lethal dose of anesthesia (100 mg/kg ketasol, Richterpharma (Austria), 50 mg/kg xylazine (Rompun, Bayer, Germany) was injected and cervical dislocation was performed for euthanasia. For the biochemical analysis of the rats, blood samples were taken by cardiac puncture method (Figure 1).

### Thorax CT examination

**Figure 2:**
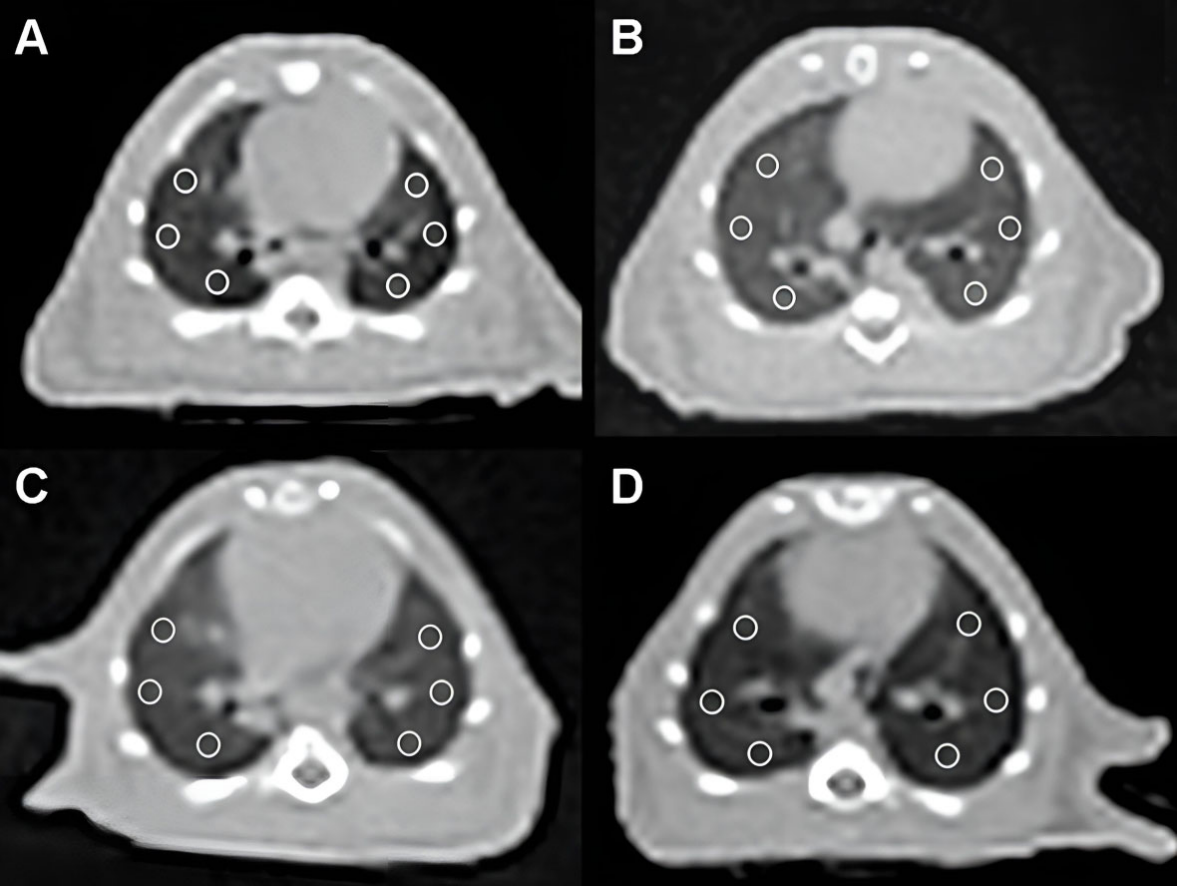
Axial computed tomography images of lung at the level of the heart. Six regions of interest (circles) with the same size were placed at the same location. **A**, Control group lung. **B**, Feces intraperitoneal procedure (FIP) group showed increased lung density. **C**, FIP + saline (10 mL/kg 0.9% NaCl) group showed increased lung density. **D**, FIP + betaine (500 mg/kg) group showed lung density similar to control group.

A CT scanner with 16-slice multi-detector row (Siemens Healthcare, Somatom Go Now, Germany) was used for acquiring images. Ketasol/xylazine anesthesia was used intraperitoneally. Contrast material was not used for imaging and all rats were properly fixed in a supine position to avoid motion artifacts. Scanning parameters were 120 kV, variable mAs, and 1-mm slice thickness. The area from the skull base to the upper abdomen, including the upper and lower segments of the lung, was included in the evaluation. After imaging, all raw data were reconstructed with KernelBr64 and 512×512 matrix size. Two radiologists (B. Özkul and F.E. Urfalı) defined and evaluated all images of the animal groups. Regions of interest (ROI) of the same size (2.267 mm^2^) for all animals were placed on axial images near the heart apex. The parenchymal window was used to place ROIs in the upper, middle, and lower regions of the lungs. ROIs were not placed in the great vessels, bones, and airways to avoid bias (Figure 2).

### Biochemical analysis

Plasma levels of TNF-α, IL-1β, IL-6, and C-reactive protein (CRP) were determined using commercially available enzyme-linked immunosorbent assay (ELISA) kits (Biosciences, Abcam, USA). All measurements were made according to the manufacturer’s specifications. Serum samples were diluted 1:2 as recommended by the manufacturer, and CRP and TNF-α were determined in pairs. Blood gas analysis was used to determine lactic acid levels.

### Measurement of MDA

The level of thiobarbituric acid reactive substances (TBARS) was used for detecting lipid oxidation by measuring MDA plasma levels as previously described by Wichterman et al. (20). The plasma sample was mixed with TBARS reagent and trichloroacetic acid and incubated at 100°C for 1 h. For 20 min, the mixture was centrifuged at 135 *g* with the samples on ice. The absorbance was read at 535 nm after centrifugation. Tetraethoxypropane was calibrated, and MDA concentrations were measured in nanometers (nM).

### Histopathological examination of lung

Lung histological sections (5 μm) obtained from all subjects were fixed with formalin and stained with hematoxylin and eosin (HE). Evaluations were made by a histologist (U.Y.), who was blinded to the study. Alveolar congestion, collection or infiltration of leukocytes in vessel walls/air spaces, bleeding, perivascular/interstitial edema, and alveolar wall thickness/hyaline membrane formation were evaluated and calculated using the histopathological lung injury score described by Kwon et al. (21). Lung injury was graded from mild to severe as follows: 1 (0-25%), 2 (26-50%), 3 (51-75%), 4 (76-100%). A digital camera (Olympus C-5050, Japan) mounted on a microscope (Olympus BX51) was used to photograph the sections.

### PaO_2_ and PaCO_2_ in arterial blood gas

At the end of all procedures, artery blood of the animals was sampled by cardiac puncture, and these samples were used for assaying arterial oxygen pressure (PaO_2_) and arterial carbon dioxide pressure (PaCO_2_) with a blood gas analyzer (Portable Rapidlab Siemens 348, Germany).

### Statistical analysis

The statistical evaluation was performed using SPSS, version 15.0 (IBM, USA). Data are reported as means±SE. The Shapiro-Wilk test was used to determine the normal or non-normal distribution of variables, one-way analysis of variance (ANOVA) was used to evaluate parametric variables, and *post hoc* Bonferroni correction was used for subgroup evaluation. P<0.05 was the statistical significance level.

## Results

### CT images of lung

Axial CT images of the lung at the level of the heart were captured to assess lung density. In the control group (Figure 2A), the lung exhibited normal density. In contrast, both the FIP group (Figure 2B) and the FIP + saline group (Figure 2C) showed increased lung density, indicating pathological changes. However, the FIP + betaine group (Figure 2D) displayed lung density closer to that of the control group, suggesting a beneficial effect of betaine in mitigating lung density abnormalities (Figure 2).

### Comparison of inflammatory biomarkers

**Figure 3:**
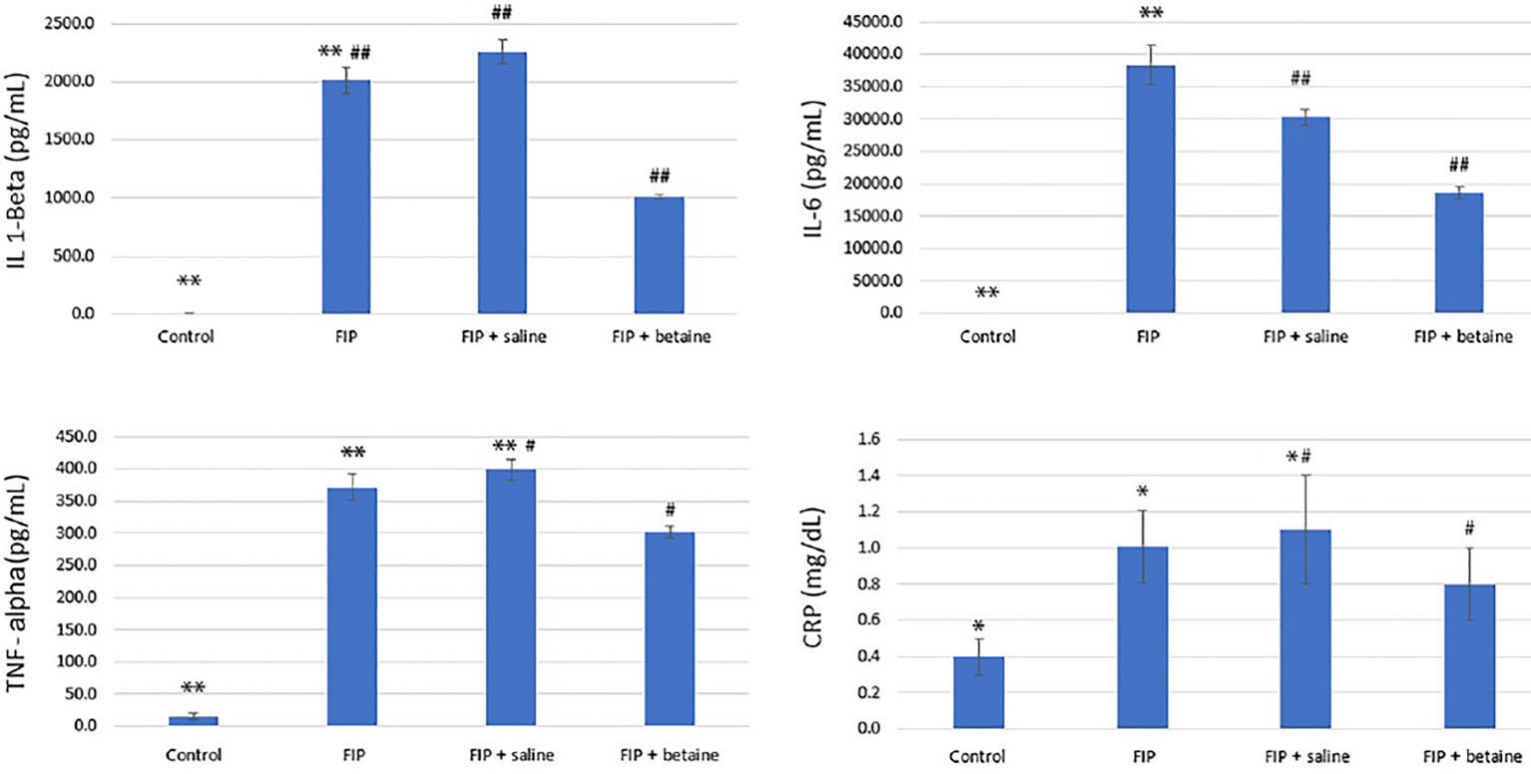
Comparison of the inflammatory biomarkers between groups: IL: interleukin; TNF: tumor necrosis factor; CRP: C-reactive protein; FIP: feces intraperitoneal injection procedure. Data are reported as mean±SE. Statistical analyses were performed by one-way ANOVA. IL 1-Beta: **P<0.001, FIP *vs* control; ^##^P<0.001, FIP + saline *vs* FIP and FIP + betaine. IL-6: **P<0.001, FIP *vs* control; ^##^P<0.0001 FIP + saline *vs* FIP + betaine. TNF-alpha: **P<0.001 FIP + saline *vs* FIP and control, ^#^P<0.01, FIP + saline *vs* FIP + betaine. CRP: *P<0.05, FIP + saline *vs* FIP and control groups, ^#^P<0.01, FIP + saline *vs* FIP + betaine.

The FIP group exhibited significantly higher levels of inflammatory biomarkers compared to the control group (P<0.05 or P<0.001). FIP + saline group did not show significant improvement in these inflammatory biomarkers compared to the FIP group. However, the FIP + betaine group displayed significant reductions in these biomarkers compared to the FIP group (P<0.01 or P<0.001) (Figure 3).

### Comparison of oxidative stress biomarkers

**Figure 4:**
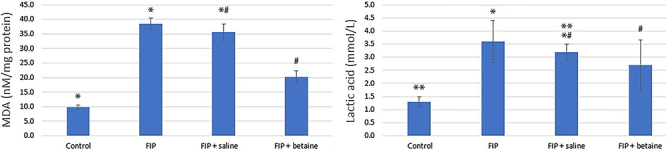
Comparison of the oxidative stress biomarkers between groups: MDA: malondialdehyde; LA: lactic acid; FIP: feces intraperitoneal injection procedure. Data are reported as means±SE. Statistical analyses were performed by one-way ANOVA. MDA: *P<0.05, FIP *vs* control and FIP + saline *vs* FIP, ^#^P<0.01 FIP + betaine vs FIP + saline. Lactic acid: **P<0.001 FIP + saline *vs* control, *P<0.05 FIP + saline *vs* FIP, ^#^P<0.01 FIP + saline *vs* FIP + betaine.

The FIP group showed significantly higher levels of oxidative stress biomarkers compared to the control group (P<0.05 or P<0.001). FIP + saline group did not significantly improve these biomarkers compared to the FIP group; however, the FIP + betaine group exhibited significant reductions in these biomarkers compared to the FIP group (P<0.01 or P<0.001). The MDA level in the FIP group was significantly higher than both the control group and the FIP + saline group (P<0.05). The MDA level was significantly higher in the FIP + saline group than the FIP + betaine group (P<0.01). The lactic acid level in the FIP + saline group was significantly higher than the FIP + betaine group (P<0.01) (Figure 4).

### Lung histopathology

**Figure 5:**
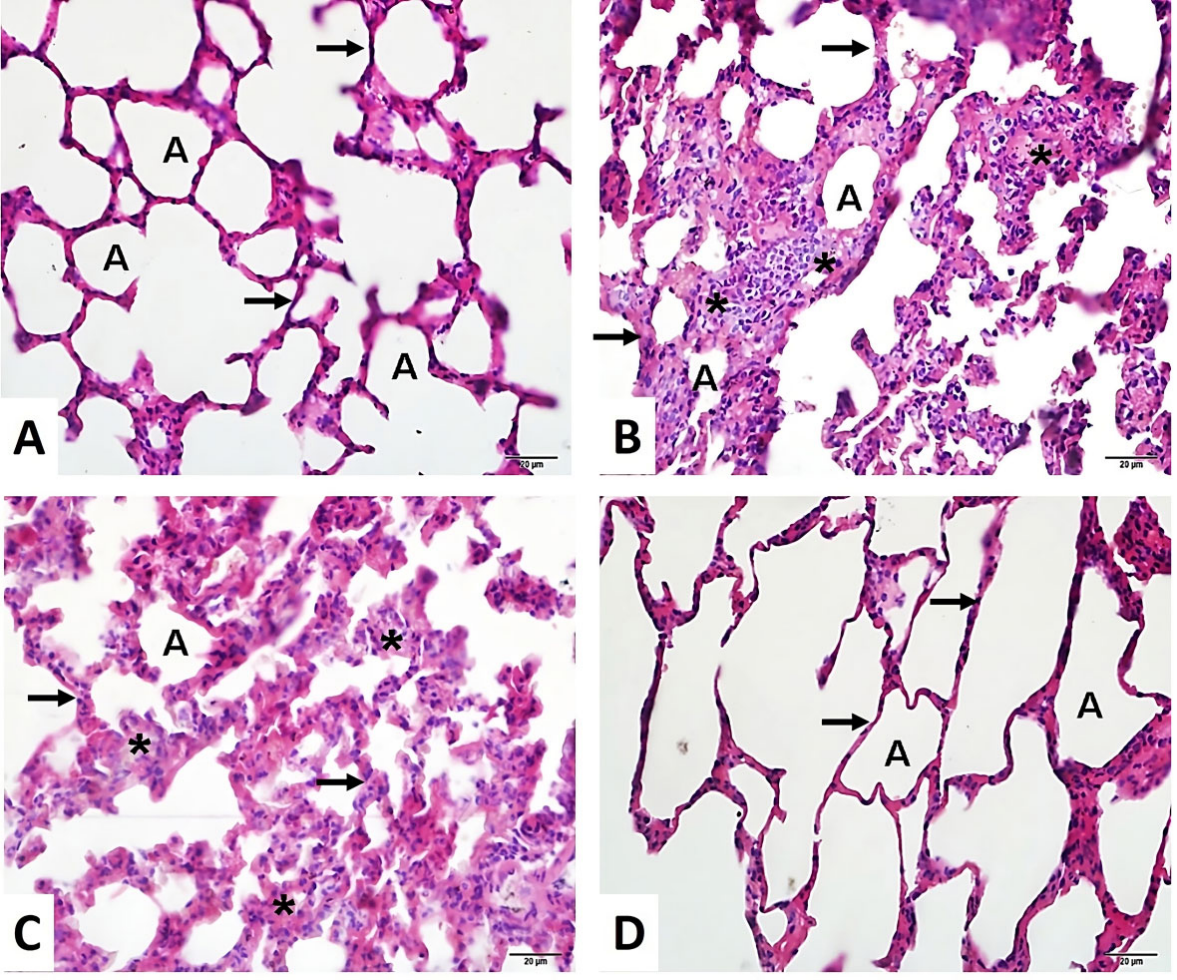
Lung histopathology (HE staining). **A**, Control group lung. **B**, Feces intraperitoneal procedure (FIP) group and **C**, FIP + saline (10 mL/kg 0.9% NaCl) group showed severe histopathologic alteration related to increased alveolar inflammation (*) and septal thickness (arrow). **D**, FIP + betaine (500 mg/kg) group showed decreased inflammation and septal thickening (arrow). The letter A in the images indicates alveoli. Magnification ×40, scale bar 20 μm.

The control group (Figure 5A) showed normal lung histology with no significant alterations. In contrast, the FIP group (Figure 5B) and the FIP + saline group (Figure 5C) displayed severe histopathological alterations, including increased alveolar inflammation and septal thickness, indicating lung injury. However, the FIP + betaine group (Figure 5D) showed decreased inflammation and septal thickening, suggesting a protective effect of betaine in lung histopathology (Figure 5).

### Histological score and CT findings

Histopathological scores and CT results are summarized in Table 1. The control group exhibited low scores for all histopathological parameters and CT low (HU) values (-632.1±14.4). In contrast, the FIP group showed significantly higher scores for all histopathological parameters and higher CT HU values (-516.9±10.8) compared to the control group (P<0.001). The FIP + saline group did not show significant improvement in histopathological scores compared to the FIP group. However, there was a slight improvement in CT HU values (-502.9±14.1) compared to the FIP group (P<0.001). On the other hand, all histopathological scores were significantly improved in the FIP + betaine group compared to the FIP group (P<0.001). Additionally, the CT HU values (-594.5±8.8) in the FIP + betaine group showed a significant improvement compared to the FIP group (P<0.05) (Table 1).

**Table 1:** Histopathological scores and computed tomography results of the groups.

	Control	FIP	FIP + saline	FIP + betaine (500 mg/kg)
AC	0.2±0.1	3.4±0.4**	3.4± 0.3*	1.1±0.1^##^
H	0.4±0.1	1.7±0.2*	2.5±0.2**	0.3±0.1^##^
AL	0.1±0.1	2.8± 0.3**	2.7±0.4**	1.2±0.2^##^
PE	0.3±0.2	3.1±0.1**	3.3±0.2**	1.8±0.3^##^
TA	0.2±0.1	2.9±0.3**	2.8±0.5**	1.7±0.3^#^
CT (HU)	-632.1±14.4	-516.9±10.8**	-502.9±14.1**	-594.5±8.8^#^

Data are reported as means±SE. Statistical analyses were performed by one-way ANOVA and *post hoc* Bonferroni correction. *P<0.01, **P<0.001 different from control group; ^#^P<0.05, ^##^P<0.001 different from FIP + saline group. AC: alveolar congestion; AL: aggregation or infiltration of leukocytes in vessel walls/air spaces; FIP: feces intraperitoneal procedure, H: hemorrhage; PE: perivascular/interstitial edema; TA: thickness of the alveolar wall/hyaline membrane formation; CT: computed tomography; HU: Hounsfield units.

### Arterial PaO_2_ and PaCO_2_

The FIP group exhibited significantly lower levels of PaO_2_ (69.8±5.6 mmHg) compared to the control group (101.2±8.8 mmHg) (P<0.05) as well as significantly lower levels of PaCO_2_ (31.2±7.3 mmHg) compared to the control group (38.5±4.7 mmHg) (P<0.05). FIP + saline did not show significant improvement in the PaO_2_ levels compared to the FIP group. There was a slight improvement in PaCO_2_ levels (28.1±3.1 mmHg) compared to the FIP group, although it did not reach statistical significance. In contrast, treatment with 500 mg/kg betaine in the FIP + betaine group significantly improved the PaO_2_ levels (88.2±9.3 mmHg) compared to the FIP group (P<0.05). There were no significant differences in PaCO_2_ levels (30.9±5.5 mmHg) between the FIP + betaine group and the FIP group (Table 2).

**Table 2:** Levels of arterial blood gas in the groups.

	Control	FIP	FIP + saline	FIP + betaine (500 mg/kg)
PaO_2_ (mmHg)	101.2±8.8	69.8±5.6*	72.9±13.5*	88.2±9.3^#^
PaCO_2_ (mmHg)	38.5±4.7	31.2±7.3*	28.1±3.1*	30.9±5.5

Data are reported as means±SE. Statistical analyses were performed by one-way ANOVA and *post hoc* Bonferroni test. *P<0.05, different from control groups; ^#^P<0.05 different from FIP + saline group. FIP: feces intraperitoneal procedure.

## Discussion

In this study, we found that betaine given by *ip* injection in the sepsis group decreased the levels of pro-inflammatory cytokines (TNF-α, CRP, IL 1-β, IL-6) and oxidative stress biomarkers (MDA, LA) and increased PaO_2_. Histopathological lung injury score and density (HU) of lung parenchyma in CT examinations were lower in rats with sepsis treated with betaine. Our study is the first in the literature to show that betaine reduced the severity of ALI with pro-inflammatory cytokines, oxidative stress biomarkers, CT, and histopathological findings.

Betaine controls the production of inflammation-related pro-inflammatory cytokines, such as NF-κB, TNF-α, and IL-1β. The NF-κB signaling pathway is an essential candidate for treating inflammation. Researchers have found that betaine can suppress NF-κB activity and various downstream genes (22,23).

In fructose-induced nonalcoholic fatty liver disease (NAFLD) models, betaine treatment has been shown to significantly inhibit levels of pro-inflammatory cytokines, including IL-1β, in a dose-independent manner (17). Xia et al. (24) reported that pro-inflammatory macrophages convert betaine to hypoxanthine and reduce IL-1β levels. On the other hand, Shi et al. (25) found that betaine decreased serum TNF-α and interferon-gamma (INF-γ) levels in rats exposed to alcohol and damaged liver parenchyma. Zhang et al. (22) showed that adding betaine to drinking water stopped the production of pro-inflammatory cytokines like IL-6 and had an anti-inflammatory effect in a chicken model of bursal inflammation. Similarly, in this study, betaine probably decreased inflammatory markers by the homocysteine methionine pathway.

Betaine administration has also shown to attenuate serum levels of IL-6 and TNF-α in rats who have inhaled hookah smoke (25). According to Zhang et al. (22), rat lungs had lower levels of betaine following the operation to create a peritonitis model. We observed a significant reduction in IL-1-β, IL-6, and TNF-α pro-inflammatory cytokines with betaine treatment in sepsis-induced rats, in line with the studies in the literature. CRP is one of the biomarkers used for sepsis diagnosis and follow-up (26). Because it is an acute phase reactant, betaine is increased in many diseases. In this study, betaine administration significantly decreased CRP. These data were consistent with the study by Vukićević et al. (27).

In the animal experiment conducted by Pourmehdi et al. (28), it was revealed that the level of IgE was also significantly reduced in asthmatic rats treated with betaine compared to asthmatic rats treated with prednisolone, and there was a marked reduction in thickening of the airway epithelium and infiltration of inflammatory cells in rats treated with betaine. Betaine can protect the liver and kidneys from oxidative stress in mice with asthma.

Oxidative stress plays a pivotal role in the pathogenesis of ALI, leading to tissue damage, activation of inflammatory cascades, and impairment of lung function. Na et al. (29) conducted animal experiments that emphasized the preventive effects of betaine on fibrotic lung changes induced by paraquat exposure and the enhancement of pulmonary antioxidant capacity in rats. The study revealed that betaine's regulation of sulfur-dependent amino acid metabolism could augment the lung's antioxidant defense mechanism against oxidative stress. Hagar et al. (30) conducted studies on cisplatin-induced nephrotoxicity and reported that betaine exhibited inhibitory effects on lipid peroxidation. Betaine acted by suppressing the activation of renal TBARS, which was initiated by oxidative stress.

In rats with sepsis, we found that betaine decreased MDA and LA levels by lowering oxidative stress. We also found that lung injury led to a drop in histopathological scores, which aligns with other studies. Because ALI makes capillaries more permeable, proteinaceous materials leak from the intravascular space into the alveolar space. This causes edema, which can be observed on CT as an increase in density and measured quantitatively from the HU value (7-11,31).

Metabolic acidosis induced by lactic acid occurs in instances of sepsis, leading to a decrease in PaCO_2_ levels. Metabolic acidosis was noted in all sepsis cohorts compared to the control cohort (32). The previous observation implies the existence of metabolic acidosis within the sepsis condition concomitant with a decline in CO_2_ concentrations as a compensatory mechanism. The presented data showed that the group that received betaine treatment exhibited persistent acidosis and that this treatment did not result in a favorable outcome concerning acidosis. Acidosis is the likely cause of mortality in the rats. The continued acidosis in live rats provided additional evidence to support the notion of this unfavorable state. The group that received betaine had higher survival rates, which could be linked to the effects of betaine on the innate immune system and antioxidant pathways.

### Conclusion

Based on CT findings, histopathological outcomes, and antioxidant and anti-inflammatory markers, betaine presented protective effects against ALI and ARDS in septic rats. However, further elucidation of the benefits of betaine requires long-term prospective studies in human subjects.
